# Opinion: Are mental health benefits of the ketogenic diet accompanied by an increased risk of cardiovascular disease?

**DOI:** 10.3389/fnut.2024.1394610

**Published:** 2024-05-01

**Authors:** David M. Diamond, Paul Mason, Benjamin T. Bikman

**Affiliations:** ^1^Department of Psychology, University of South Florida, Tampa, FL, United States; ^2^Orthosports, Concord, NSW, Australia; ^3^Department of Cell Biology and Physiology, Brigham Young University, Provo, UT, United States

**Keywords:** ketogenic diet, low carbohydrate diet, cardiovascular disease, neurological disorder, cholesterol, low-density lipoprotein (LDL), risk factor

## Introduction

Ketogenic (very low carbohydrate) diets have well-established, as well as potential, benefits in the treatment of neurological disorders. Over a century ago the ketogenic diet was adopted as an effective treatment for epilepsy (1). More recently, ketogenic diets have demonstrated promising therapeutic potential in a broad range of neurological disorders, including Alzheimer's disease, Parkinson's disease, multiple sclerosis, ischemic stroke, migraine, major depressive disorder, bipolar disorder and psychotic illness (2–5), as well as a potential treatment for traumatic brain injury (6). This research has identified great promise in the use of the ketogenic diet to improve brain functioning, particularly in response to psychiatric disorders and injury.

The ketogenic diet, however, is not without its detractors. A concern with the ketogenic diet is that in some individuals very low carbohydrate consumption can lead to dramatic increases in the level of low-density lipoprotein cholesterol (LDL-C) (7, 8), which is considered a primary cause of cardiovascular disease (CVD) (9). Whereas the ketogenic diet is beneficial for mental health and in the treatment of neurological disorders, but for some individuals with elevated LDL-C, is that benefit obtained at the cost of increasing their risk of developing CVD? We have addressed this issue with an analysis of the benefits vs. potential harms of a ketogenic diet-induced increase in LDL-C.

## Is elevated LDL-C inherently atherogenic?

An elevated level of LDL-C has been described as “unequivocally recognized as the principal driving force in the development of (atherosclerotic cardiovascular disease)” (9) and that “the key initiating event in atherogenesis is the retention of low-density lipoprotein (LDL) cholesterol (LDL-C) … within the arterial wall” (10). The view that high LDL-C is atherogenic provides the basis for why an LCD-induced increase in LDL-C has been seen as increasing the risk for developing CVD (8, 11–18). In one example, a ketogenic diet-induced increase in LDL-C was the topic of an editorial that stated these individuals should “work closely with their doctor to implement lifestyle changes and/or medical therapy directed toward lipid lowering with the aim of reducing cardiovascular risk” (18).

Although LDL-C as a cause of CVD is the consensus of key opinion leaders, there are findings that are not supportive of this perspective. An inconsistent, and largely ignored, finding is that cardiovascular and all-cause mortality in people with familial hypercholesterolemia (FH), who have extremely high levels of LDL-C from birth, declines with advanced age, resulting in an overall normal lifespan (19–23). Moreover, people with FH exhibit an equivalent degree of aspects of cardiovascular morbidity, such as ischemic stroke (24), as the general population. These findings challenge the consensus that high LDL-C is inherently atherogenic.

What has been largely ignored in the consensus opinion of FH is that only a subset of individuals with FH die prematurely of CVD. A close assessment of this research reveals that this subset of FH individuals develop coagulopathy, independent of their LDL-C levels (25–29). In one representative study, Jansen et al. (28) reported that FH patients that developed CVD had a polymorphism for the prothrombin gene, which is also associated with premature CVD in the non-FH population (30). Sugrue et al. (31), as well, reported that FH individuals with coronary heart disease (CHD) had higher levels of clotting factors (plasma fibrinogen and factor VIII), and conversely, Sebestjen et al. (32) found reduced markers of fibrinolysis in FH individuals that experienced a myocardial infarction, both of which were independent of their LDL-C.

In complementary research, high LDL-C appears to protect against bacterial infection, which is a risk factor for CVD (33–39). The protection of individuals with high LDL-C from infection and its sequalae is manifested, in one example, by the significantly lower rate of sepsis, and sepsis-induced organ damage, in people with high LDL-C, compared to those with low LDL-C (40).

**Figure 1:**
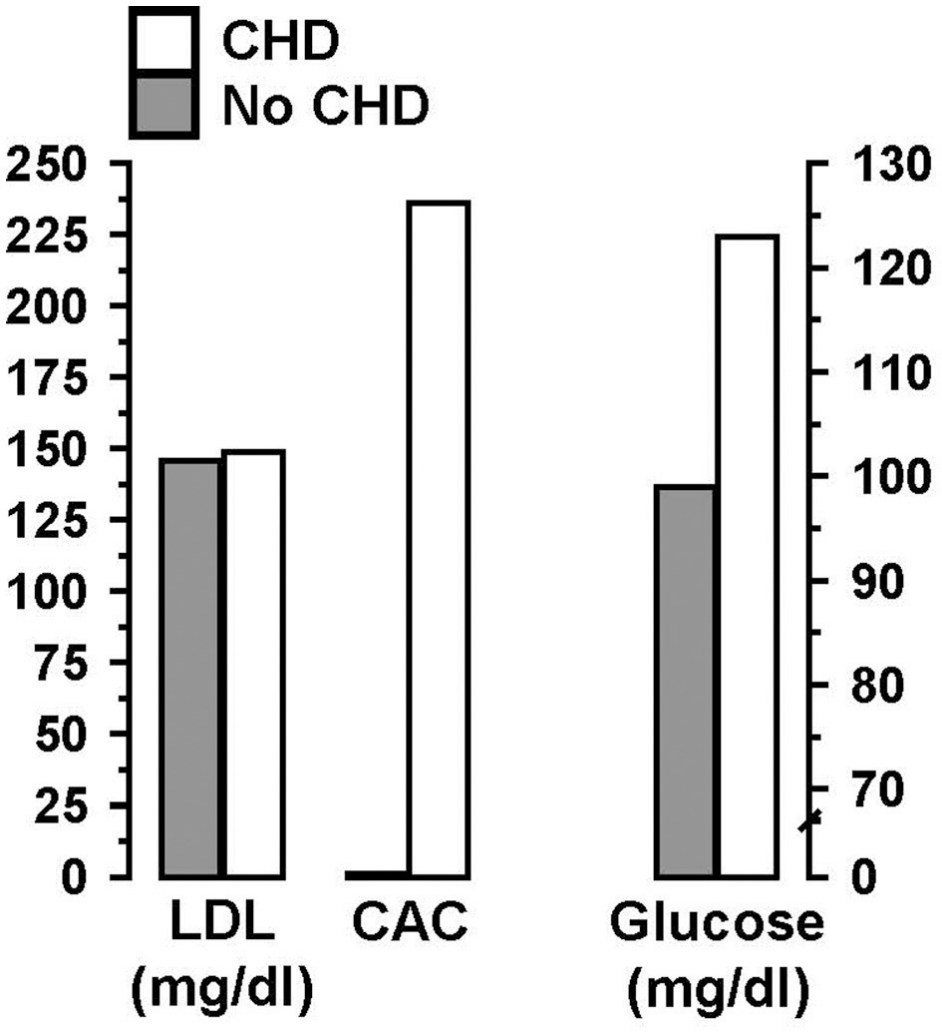
In individuals with familial hypercholesterolemia (FH), a high coronary artery calcium (CAC) score and elevated fasting glucose, unlike high low-density lipoprotein cholesterol (LDL-C), were associated with coronary heart disease (CHD). Data from Miname et al. (45).

With regard to the critical factors leading to CVD susceptibility, it has long been recognized that coronary artery calcium (CAC) scoring is superior to LDL-C as the single best predictor of fatal and non-fatal coronary events (41–44). For example, approximately half of FH individuals assessed showed zero CAC, which would indicate they have a low risk for developing CVD, despite their high LDL-C levels (45). Moreover, this study demonstrated that a high CAC score and elevated fasting glucose, unlike LDL-C, were both associated with coronary events (Figure 1). Similar findings were reported by Mortensen et al. (46) in a study of non-FH individuals. These findings led Bittencourt et al. (47), to conclude that “treatment of individuals with very high LDL-C (>190 mg/dl) irrespective of their clinical risk … might not be the most prudent approach.”

At a mechanistic level, concerns with a ketogenic diet-induced increase in LDL-C have not taken into account that the “total LDL-C” measure reported in a conventional lipid panel represents a heterogeneous population of different LDL particle types (48, 49), one of which is referred to as lipoprotein (a) [Lp(a)]. An elevation of Lp(a) is an independent risk factor for the development of CVD (50–54). The association of Lp(a) to CVD may be driven, in part, by its strong atherogenic effects at multiple metabolism levels, particularly in promoting thrombosis (55, 56). For example, Yang et al. (57) demonstrated that the combination of high Lp(a) and fibrinogen levels were correlated with the highest incidence of ischemic stroke in statin-treated patients, while LDL-C levels were unrelated to stroke incidence. Finally, Willeit et al. (58) showed that Lp(a) is a critical component of the association of LDL-C with CVD; without the Lp(a)component, LDL-C, alone, was not associated with CVD.

## Insulin resistance and cardiovascular disease

Hyperinsulinemia and hyperglycemia, collectively referred to as insulin resistance (IR), are strong and independent risk factors for CVD (59–63). IR may develop into type 2 diabetes, which typically is not accompanied by an elevation of LDL-C (64), and yet it has the greatest risk for CVD (65). There are multiple mechanisms by which IR exerts an adverse effect on blood vessel structure and functioning leading to CVD (60, 61, 66–71). For example, Yu et al. (72) reported that elevated fasting plasma glucose, hemoglobin A1c and triglycerides (TG), unlike, LDL-C, were all positively correlated with the severity of coronary stenosis. Thus, IR is superior to LDL-C as a marker for CVD risk.

**Figure 2:**
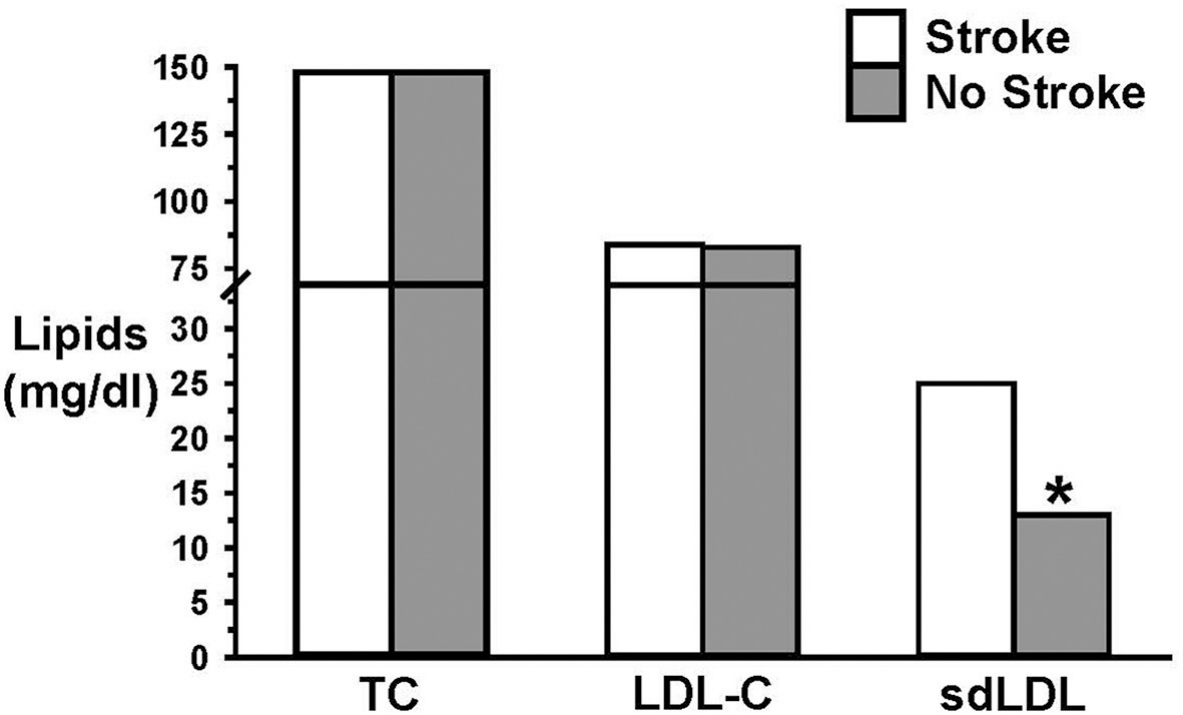
Elevated small dense LDL (sdLDL), but not LDL-C, was an independent risk factor for ischemic stroke. Data from Zhou et al. (87). * = *p* < 0.05.

An important but often ignored influence on LDL-C structure and function is referred to as atherogenic dyslipidemia, in which elevated LDL-C is accompanied by elevated triglycerides and low HDL, which is a common metabolic state in people with Type 2 diabetes and obesity (73–75). Under atherogenic dyslipidemia conditions, the composition of the LDL particles (LDL-P) exhibits a shift toward a greater density of small, dense LDL-P (sdLDL) and a reduced density of large, buoyant LDL-P (lbLDL). This shift in the dominance of sdLDL over lbLDL is characteristic of a pro-atherogenic state, originally described as “phenotype B” (76). Phenotype B, in contrast to those with low triglycerides, high lbLDL and high HDL (phenotype A), is strongly associated with an increased incidence of CVD (48, 56, 77–90). One example of this finding is that an elevated level of sdLDL, but not LDL-C or lbLDL, was an independent risk factor for ischemic stroke (87) (Figure 2). Numerous observational studies, as well, have shown that lbLDL is not associated with CVD (91–94).

It is therefore important to recognize that the primary reason why LDL-C is a poor marker for CVD risk is because it is a hybrid measure, composed of different sizes of LDL particles (sdLDL and lbLDL), as well as Lp(a) (discussed previously), each with a different association to metabolic health and CVD risk (90, 95) [see also Gjuladin-Hellon et al. (96) and Diamond et al. (97) for related review and discussion].

## Effects of low carbohydrate diets on cardiovascular disease risk factors

Carbohydrate restriction has been shown to improve a broad range of CVD risk factors (49, 98–122). It is notable that along with the improvement in metabolic measures, LCD reduces the need for hypoglycemic and antihypertensive medications (111, 123–132). Moreover, LCDs attenuate the atherogenic dyslipidemia risk triad (reducing TGs, sdLDL, increasing lbLDL and HDL) (49, 96, 105, 133–136). Long-term trials and case reports have demonstrated the benefits of LCD (49, 100, 102, 137–144) and in documenting improvements in numerous CVD risk biomarkers (133, 144–146).

Despite the improvements in CVD risk factors with LCD, there remain concerns about LCD because of the absence of research on individuals with diet-induced high LDL-C and coronary events. A case study on a father and son diagnosed with FH may be of value in appreciating how atherogenic dyslipidemia is expressed as CVD risk, indirectly in relation to LCD. In this study, a father and son shared the same LDL mutation which resulted in both being diagnosed with FH. Despite their equivalently high levels of total cholesterol (344 vs. 352 mg/dl; father vs. son) and LDL-C (267 vs. 271 mg/dl; father vs. son), only the son (54 years old), but not the father (84 years old), had coronary heart disease (CHD). Although dietary assessments were not provided, the authors suggested that differences in their lifestyles and diets may have been a contributing factor to their differential incidence of CHD, independent of their LDL-C. Specifically, the father's triglycerides at 124.0 mg/dl were almost half of the 230.0 mg/dl measured in his son, and the father's HDL at 54.0 mg/dl was far greater than his son's HDL at 34.8. Thus, the high triglycerides and low HDL of the son provided the basis of the authors' perspective that the son exhibited LDL subclass pattern B, which is associated with a high risk of CVD and a high carbohydrate diet (75, 76). Overall, these findings are consistent with the work of Sijbrands et al. (22), who concluded that cardiovascular outcomes in people with FH are not determined solely by high LDL-C, and instead are the result of the interactions among lipids, genetics and dietary factors.

## Discussion

We have addressed concerns regarding high LDL-C that can develop in a subset of individuals on a ketogenic diet. Our commentary has evaluated whether these concerns are justified. We have briefly summarized research which has demonstrated that LDL-C is a faulty marker of CVD risk because it is a hybrid measure composed of multiple components, each with a different association to CVD. Specifically, LDL-C includes lbLDL, sdLDL, and Lp(a), each of which can be influenced by proximal influences on CVD, such as insulin resistance, hypertension, hyperglycemia and more generally, metabolic syndrome. Thus, sdLDL and Lp(a) are not intrinsically atherogenic; each becomes an atherogenic component of the maelstrom of metabolic dysfunction that occurs in response to metabolic syndrome.

The component of LDL-C that dominates in metabolically healthy people is the lbLDL particle, which is not associated with CVD events. Observational trials and RCTs have demonstrated that individuals with high LDL-C and a dominance of lbLDL (phenotype pattern A) and an LCD-like lipid profile (low TGs and high HDL-C), have a lower rate of coronary events than those with pattern B (high LDL-C, high TGs, and low HDL-C) (147, 148).

In summary, our review of the literature provides support for the conclusion that elevated LDL-C occurring in an individual on a ketogenic diet does not place a person at an elevated risk for CVD. Indeed, a person on a ketogenic diet would exhibit a dominance of beneficial lipid markers (low triglycerides, high HDL, high lbLDL), as well as beneficial non-lipid markers (low inflammation, blood glucose, and blood pressure). These findings support the conclusion that pharmacological or dietary interventions to reduce LDL-C in an individual on LCD are not warranted. Indeed, this favorable cluster of LCD-induced changes in biomarkers should not only result in a reduced risk of CVD, it should promote beneficial health outcomes based on the important role of LDL in optimizing immune functioning.

## Author contributions

DD: Writing – original draft, Writing – review & editing. PM: Writing – review & editing. BB: Writing – review & editing.
